# General Practitioners and the Out-Patient Department

**Published:** 1908-05-09

**Authors:** 


### General Practitioners and the Out-Patient Department.

We would especially draw the attention of our readers to the full report of a recent important meeting of the medical practitioners of South London, which appears in the present number of The Hospital. The meeting on Thursday, April 30, was convened by the South London Hospitals Committee, a body consisting of twelve medical men elected five years ago to represent the members of the medical profession resident in this large district. The object of the meeting was to discuss the course of action to be adopted by the local practitioners in the matter of the forthcoming removal of King's College Hospital to Denmark Hill. The two main questions arising out of the removal of the hospital were the admission of paying patients into the wards of general hospitals, and the relation of hospital out-patient departments to the medical practitioners resident in their neighbourhood. The proposed establishment in Camberwell of a large general hospital, with a large out-patient department, has, of course, for some time past received the close consideration of the South London practitioners, since the change cannot fail to affect their interests. Local medical opinion has been strongly opposed to the introduction into this district of a large out-patient department, conducted on the usual lines of the great London hospitals; and a scheme has been proposed by the South London Hospitals Committee for limiting the number of out-patients by a system of certificates to be issued by local practitioners.

We gather that the members of the committee, and, therefore, also those whom they represent, do not seek to oppose the coming of King's College Hospital to South London, but rather are prepared to welcome its advent, provided that it involves no risk of injury to local medical interests. This is an attitude which can well be understood. By their appointment of a Watching Committee (without executive powers) the authorities of King's College Hospital have shown that they are not indifferent to the interests and the point of view of local medical men, and that they recognise that the removal of the Hospital is an undertaking which involves many large issues, and will affect to a profound degree the welfare not only of the Hospital itself and all now connected with it, but also of the poor of South London, and of a large section of the medical profession. These two committees have conferred together in all friendliness, and the recommendations of the local medical representatives have for the most part been endorsed by the Watching Committee. There is, however, a difference of opinion between the two committees upon the question of the admission of paying patients into the general wards, and the sense of the recent meeting was strongly opposed to this measure. We have referred before to this point, and have given the reasons why, under proper safeguards, the paying system should be upheld. With regard to the general scheme for the limitation of the out-patient department of the new King's College Hospital and the prevention of the abuse of charity, we may point out that the proposals of the South London Hospitals Committee are precisely along the lines which have been laid down in this journal, more especially in a leading article on April 18, 1908, wherein was written: "The real remedy for the existing abuses lies in the conversion of the out-patient department into an auxiliary to, as opposed to a competitor with, the doctor, by using it for consultation purposes only for suitable cases." Should the first sixteen recommendations of the local committee, which, with one exception, have been approved by the Watching Committee, be adopted by the various hospital authorities, the administration of the out-patient department of King's College Hospital on its removal to South London should go far towards solving the great problem of the abuse of charity. If the methods suggested, with such modifications as may be necessary, are found to be practicable, other hospitals may sooner or later be compelled to follow suit, and the differences now existing between hospitals and general practitioners should be settled.

